# An acetyl-L-carnitine switch on mitochondrial dysfunction and rescue in the metabolomics study on aluminum oxide nanoparticles

**DOI:** 10.1186/s12989-016-0115-y

**Published:** 2016-01-16

**Authors:** Xiaobo Li, Chengcheng Zhang, Xin Zhang, Shizhi Wang, Qingtao Meng, Shenshen Wu, Hongbao Yang, Yankai Xia, Rui Chen

**Affiliations:** 1 Key Laboratory of Environmental Medicine Engineering, Ministry of Education, School of Public Health, Southeast University, Dingjiaqiao 87, Nanjing, 210009 China; 2 Center for Drug Safety Evaluation and Research, China Pharmaceutical University, Nanjing, 211198 China; 3 Key Laboratory of Modern Toxicology of Ministry of Education, School of Public Health, Nanjing Medical University, Nanjing, 211166 China; 4 State Key Laboratory of Bioelectronics, Southeast University, Nanjing, 210096 China

**Keywords:** Aluminum oxide nanoparticles, Mitochondria, Acetyl-L-carnitine, Nanotoxicology, Metabolomics

## Abstract

**Background:**

Due to the wide application of engineered aluminum oxide nanoparticles and increased aluminum containing particulate matter suspending in air, exposure of human to nano-scale aluminum oxide nanoparticles (Al_2_O_3_ NPs) is becoming inevitable.

**Methods:**

In the present study, RNA microarray coupled with metabolomics analysis were used to uncover mechanisms underlying cellular responses to Al_2_O_3_ NPs and imply the potential rescue.

**Results:**

We found that Al_2_O_3_ NPs significantly triggered down-regulation of mitochondria-related genes located in complex I, IV and V, which were involved in oxidative phosphorylation and neural degeneration pathways, in human bronchial epithelial (HBE) cells. Subsequent cell- and animal- based assays confirmed that Al_2_O_3_ NPs caused mitochondria-dependent apoptosis and oxidative stress either in vitro or in vivo, which were consistent with the trends of gene regulation. To rescue the Al_2_O_3_ NPs induced mitochondria dysfunction, disruption of small molecular metabolites of HBE were profiled using metabolomics analysis, which facilitates identification of potential antagonizer or supplement against nanoparticle-involved damages. Supplementation of an antioxidant, acetyl-L-carnitine, completely or partially restored the Al_2_O_3_ NPs modulated gene expression levels in mitochondrial complex I, IV and V. It further reduced apoptosis and oxidative damages in both Al_2_O_3_ NPs treated HBE cells and animal lung tissues.

**Conclusion:**

Thus, our results demonstrate the potential mechanism of respiratory system damages induced by Al_2_O_3_ NPs. Meanwhile, based on the metabolomics profiling, application of acetyl-L-carnitine is suggested to ameliorate mitochondria dysfunction associated with Al_2_O_3_ NPs.

**Electronic supplementary material:**

The online version of this article (doi:10.1186/s12989-016-0115-y) contains supplementary material, which is available to authorized users.

## Background

Nano-scale aluminum oxide (Al_2_O_3_) particles are widely used in insulator layers, powder coating, and fluorescent lamp material. Meanwhile, aluminum (Al) is a common metal component in ultrafine airborne particles (PM_2.5_) in the ambient environment^1,2^ and is relatively stable in the form of aluminum oxide. These ultrafine aluminum particles have different physical, chemical, and biological characteristics, and are likely to be more toxic than the same conventional sized materials [1]. Thus, increased and inevitable occupational and environmental exposures to ultrafine Al_2_O_3_ particles are considered as a health risk, while, prevention or intervention measures have not been investigated. Generally, ultrafine particles can be inhaled more deeply than large particles, leaving sediment on the surface of the trachea, bronchus, and alveoli. Lung tissue is considered the primary target organ for inhaled ultrafine particles. Accordingly, aluminum oxide nanoparticles (Al_2_O_3_ NPs) were employed as an ultrafine particle model to explore the impacts of aluminum ultrafine particles on the respiratory system.

Apoptosis is an endpoint to assess toxicity of metal oxide nanomaterial, providing a benchmark against which to evaluate the potential toxicity of engineered nanoparticles [2–4]. There are two major apoptotic pathways initiated by either mitochondria or cell surface receptors [5]. Mitochondria-mediated apoptosis is also called the ‘intrinsic’ pathway and occurs in response to a wide range of death stimuli, and is characterized by cascade reactions including mitochondrial outer membrane permeabilization (MOMP), release of cytochrome *c* into the cytoplasm to activate caspase-9 and subsequent caspase-3 [5, 6]. The intrinsic pathway is involved in immune disorders [7], neurodegeneration [8] and cancer [9, 10]. Studies investigating ambient particulate matter or nanoparticles suggest apoptosis or mitochondrial dysfunction are sufficient end-points to monitor toxicity [11–13], but conventional toxicity assays may not suffice to fully understand the cellular responses of ultrafine particle exposure. Thus, a more comprehensive approach to determining how cells respond to ultrafine particles is required.

“Omics” analysis, including transcriptomics, proteomics, and metabolomics, coupled with appropriate computational approaches to determine statistically significant gene, protein, metabolite or pathway regulation can be used as a tool to identify the potential hazards and mechanisms of nanoparticle toxicology [14–16]. These newly developed high-throughput approaches have been used to study the impact of nanomaterials, including metal and metal oxide nanoparticles [17–19]. In such studies, the “omics” technologies are used to predict interaction between nanoparticles and biological systems, facilitate assessment of systemic toxicity due to nanomaterials, reveal potential strategies for risk mitigation.

Metabolomics analysis is one of the most applied “omics” analysis that facilitates understanding of the modulation of small molecules following exposure. Acetyl-L-carnitine (ALCAR), an antioxidant dietary supplement, could be detected through GC/TOF/MS analysis and plays a vital role in oxidation of fatty acid metabolic pathways. It is a constituent of the inner mitochondrial membrane and has many fundamental functions, including acetyl CoA uptake [20], improving mitochondrial bioenergetics [21] and prevention of mitochondrial enzyme oxidation [22]. The acetyl group of ALCAR is used to produce the antioxidant glutathione (GSH), reducing oxidative stress, and protecting cells against lipid peroxidation [23, 24]. ALCAR also contributes to the bioenergetics processes, therefore, it plays a vital role in mitochondrial-related disorders [25, 26].

Here, we used human bronchial epithelia (HBE) as a model system due to their importance in defense against inhaled pathogens and particulates [27]. We investigated changes in gene expression profiles and small molecular metabolites in HBE cells induced by Al_2_O_3_ NPs. Gene Ontology (GO) and the Kyoto Encyclopedia of Genes and Genomes (KEGG) were used to evaluate pathways. We showed that Al_2_O_3_ NPs are capable of triggering specific changes in HBE cell gene expression, specifically in mitochondria associated genes. We assessed mitochondria-mediated apoptosis in the presence or absence of acetyl-L-carnitine as a supplement to oppose mitochondrial dysfunction caused by Al_2_O_3_ NPs. The pathological alterations and ALCAR rescue were observed in mouse lung tissues treated with Al_2_O_3_ NPs. Our results suggests Al_2_O_3_ NPs cause toxicity and mitochondrial dysfunction that may be remedied by acetyl-L-carnitine treatment.

## Results

### Overview of mRNA microarray profiles

**Fig. 1:**
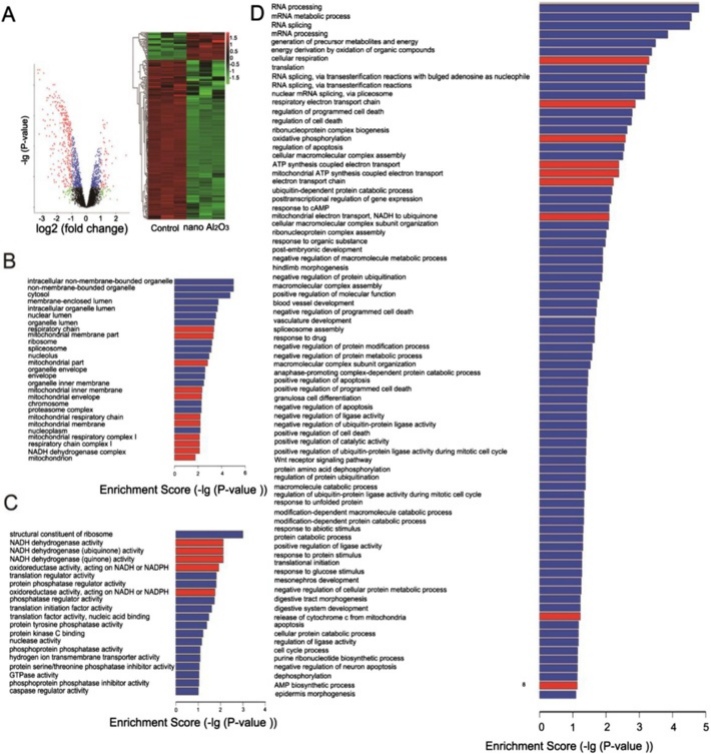
An overview of RNA microarray profiles. **a** Volcano diagram depicting the differentially expressed genes identified by a linear model of *limma* Software Package. Each red point corresponds to one differentially expressed gene. Heat map of samples based on the RNA microarray gene expression profiles. The down-regulation and up-regulation of genes were expressed by green color and red color correspondingly. **b** sub-ontology cellular component (CC) of gene ontology enrichment **c**) the sub-ontology molecular function (MF) shows mitochondria are the mostly involved enrichment categories. **d** sub-ontology of biological process (BP). The mitochondria-invovled GO enrichments were labeled with red

To determine how gene transcription was altered due to Al_2_O_3_ NP exposure, HBE cell mRNA profiling was evaluated by RNA microarray. As shown in Fig. 1a, Al_2_O_3_ nanoparticles exerted specific transcriptional effects leading to significantly increased expression of 54 genes and decreased expression of 304 genes, with a cutoff as 2 or more fold change and *P* < 0.05. GO enrichment classifies transcriptional changes observed between control and treatment groups. Notably, the GO analysis revealed that genes encoding proteins necessary for mitochondrial function were differentially expressed. Significantly, of the genes differentially regulated, 23/ 154 genes involved in cell components (CC) (Fig. 1b); 11/85 genes involved in molecular function (MF) (Fig. 1c), and 14/183 in biological process (Fig. 1d). These mitochondria involved enrichment were labeled with red color in Fig. 1. Therefore, a total of 27 mitochondrial related genes were identified, the fold change, *P* value and functions of their encoded proteins are shown in Table 1.

**Table 1:** Modulated genes involved in mitochondrial functions

Gene symbol	Main functions	Adjusted *P* value	Fold change
NDUFA4	NADH dehydrogenase [ubiquinone] 1 alpha subcomplex subunit 4	6.73E-06	2.365756
NDUFA2	NADH dehydrogenase [ubiquinone] 1 alpha subcomplex subunit 2 isoform 1	3.02E-06	0.374527
NDUFS4	NADH dehydrogenase [ubiquinone] iron-sulfur protein 4, mitochondrial precursor	4.13E-06	0.396259
UQCR11	ubiquinol-cytochrome c reductase, complex III subunit XI [Source:HGNC Symbol;Acc:30862]	4.01E-06	2.23789
COX7B	cytochrome c oxidase subunit 7B, mitochondrial precursor	2.16E-07	0.284784
NDUFC2	NADH dehydrogenase [ubiquinone] 1 subunit C2 isoform 1	4.23E-07	0.262135
NDUFA1	NADH dehydrogenase [ubiquinone] 1 alpha subcomplex subunit 1	3.91E-08	0.217706
ATP5H	ATP synthase subunit d, mitochondrial isoform b	3.12E-06	0.400955
TXNDC17	thioredoxin domain-containing protein 17	9.85E-03	2.426058
ATP5S	ATP synthase subunit s, mitochondrial isoform b precursor	6.20E-03	0.273283
ATP6V0B	V-type proton ATPase 21 kDa proteolipid subunit isoform 2	1.34E-03	0.450262
CS	citrate synthase, mitochondrial precursor	7.27E-05	0.464647
DLST	dihydrolipoyllysine-residue succinyltransferase component of 2-oxoglutarate dehydrogenase complex, mitochondrial isoform 1 precursor	4.25E-04	0.486408
JUN	transcription factor AP-1	8.43E-04	0.447503
NDUFB11	NADH dehydrogenase [ubiquinone] 1 beta subcomplex subunit 11, mitochondrial isoform 1	2.57E-02	2.051124
SOD2	superoxide dismutase [Mn], mitochondrial isoform A precursor	6.44E-06	0.476653
TP73	tumor protein p73 isoform d	2.15E-02	0.403954
ATPIF1	ATPase inhibitor, mitochondrial isoform 1 precursor	8.80E-04	2.074952
COX17	cytochrome c oxidase copper chaperone	6.79E-08	0.255958
MRPL11	39S ribosomal protein L11, mitochondrial isoform a	4.50E-06	0.159154
PMPCB	mitochondrial-processing peptidase subunit beta precursor	4.50E-06	0.352621
CYP11A1	cholesterol side-chain cleavage enzyme, mitochondrial isoform a precursor	4.45E-05	2.955856
MRPL52	mitochondrial ribosomal protein L52	2.41E-05	0.452092
HAAO	3-hydroxyanthranilate 3,4-dioxygenase	1.34E-03	0.376486
TOMM20	mitochondrial import receptor subunit TOM20 homolog	6.86E-07	0.321764
MRPL39	39S ribosomal protein L39, mitochondrial isoform b	1.16E-06	0.38445
HSPE1	10 kDa heat shock protein, mitochondrial	2.73E-08	0.239258

**Fig. 2:**
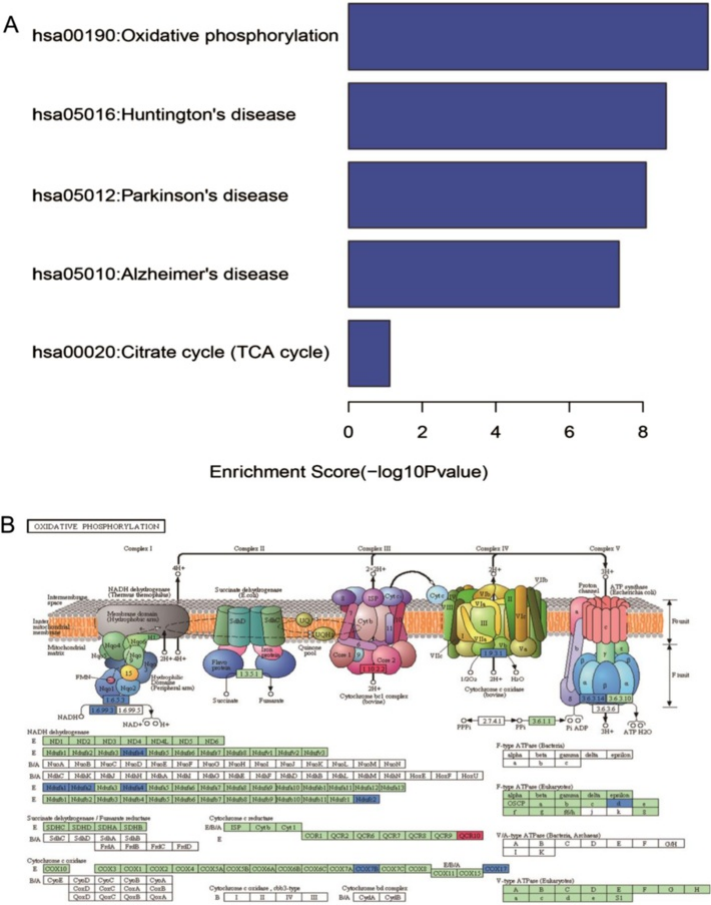
KEGG pathway enrichment analysis of mitochondria related genes. **a** A total of 27 mitochondria related genes were analyzed through DAVID functional annotation cluster tool. These genes mainly are involved in five KEGG pathways. Oxidative phosphorylation is the most significant enrichment. **b** A schematic figure of the oxidative phosphorylation pathway by KEGG. mRNA microarray assay predicted up-regulated genes are stainded red, and down-regulated genes are stained blue in this schematic figure

Subsequently, these 27 genes were subjected to KEGG pathway enrichment analysis. These selected genes were annotated with the KEGG pathway database and hypergeometric tests were used to estimate the significance of enrichment. Among these pathways, mitochondrial function and neural system disease were significantly enriched (Fig. 2a). Figure 2b is schematic oxidative phosphorylation pathway by KEGG. Our microarray data suggested that NADH ubiquinone oxidoreductase-encoding genes (NDUFA2 and NDUFS4), NADH dehydrogenase-encoding genes located in mitochondrial complex 1 (NDUFC2, NDUFA1, and NDUFA4), NADH ubiquinol-cytochrome *c* reductase gene UQCR11 (complex III), cytochrome *c* oxidase genes (COX7B and COX17 (complex IV)), and the mitochondrial ATP synthase gene ATP5H (complex V, F0 unit) were among the most affected genes. The down-regulated genes were noted with blue color and the up-regulated gene was noted with red color in Fig. 2b.

### Levels of mRNA expression were confirmed by qRT-PCR

**Fig. 3:**
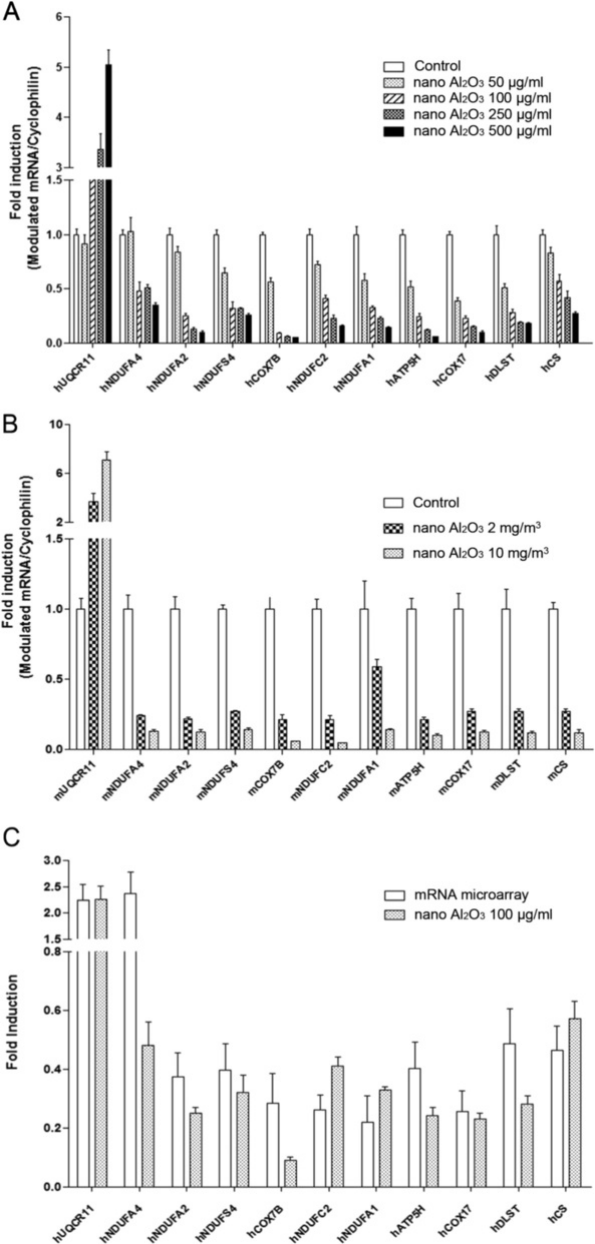
qRT-PCR validation of the expression levels of modulated mitochondria-related mRNA. **a** Mitochondria-related gene expression level in HBE cells treated with 50, 100, 250 or 500 μg/ml Al_2_O_3_ NPs. Gene expression is regulated in a dose-dependent manner **b**: Gene expression level of lung tissues in mice exposed to Al_2_O_3_ NPs through inhalation. Gene expression is regulated in a dose-dependent manner. **c** Except NDUFA4, fold change of modulated genes in cells treated with 100 μg/ml Al_2_O_3_ NPs are consistent with array data

To validate our RNA microarray data, we performed qRT-PCR to validate 11 differentially expressed mitochondrial genes that involved in at least two enrichments in GO categories. In HBE cells and lung tissue obtained from mice treated with Al_2_O_3_ NPs, UQCR11 was upregulated and NDUFA4, NDUFA2, NDUFS4, COX7B, NDUFC2, NDUFA1, ATP5H, COX17, DLST, and CS were downregulated in a dose-dependent manner (Fig. 3a and b). With the exception of NDUFA4, which was down-regulated by qRT-PCR analysis, the trend in mRNA regulation of the other 10 genes was consistent between microarray and qRT-PCR (Fig. 3c).

### Apoptosis is induced in HBE cells and lung tissue following Al_2_O_3_ NPs exposure

**Fig. 4:**
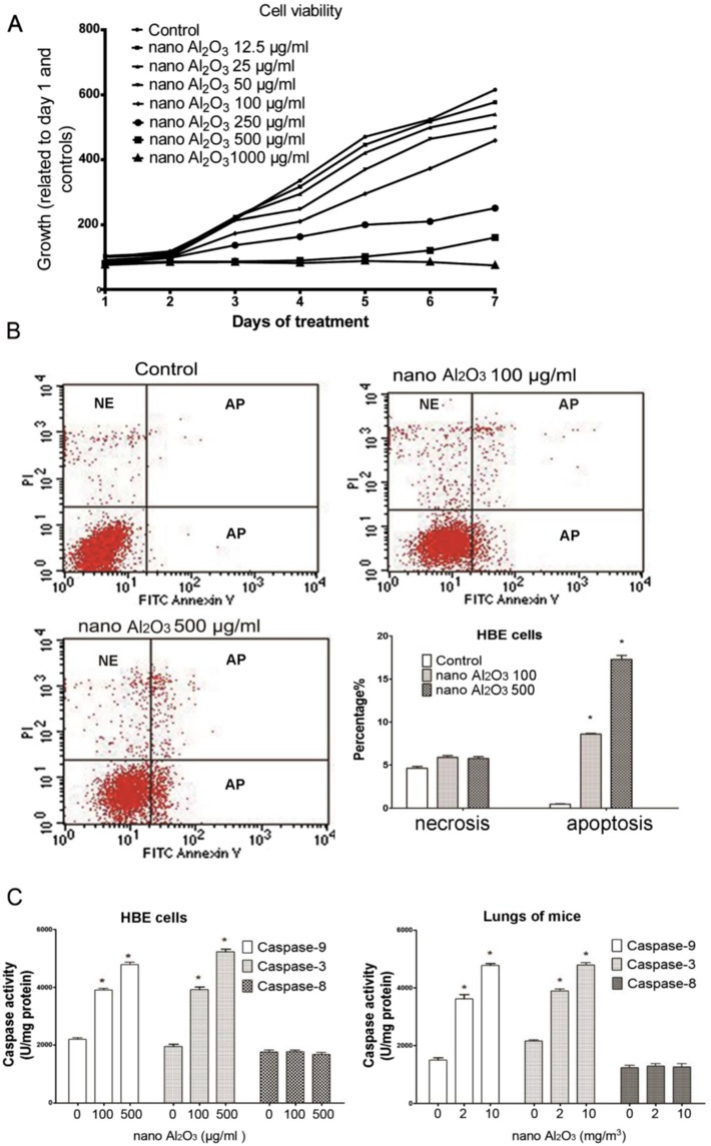
Cell viability and apoptosis of HBE cells after exposure to Al_2_O_3_ NPs. **a** Cell viability of HBE cells were analyzed through CCK-8 assays after exposure to Al_2_O_3_ NPs. **b** Apoptosis induction was significantly enhanced by Al_2_O_3_ NP exposure, and the proportion of necrosis was not significantly modulated **c**) Assessments of caspase-3, -8, and -9 activities in HBE cells and lung tissues harvested from mice treated with Al_2_O_3_ NPs. The increase of caspase-9 and -3 suggested that Al_2_O_3_ NPs induced apoptosis in HBE are mitochondria involved. ^*^ *P* < 0.05, compared with untreated control

Cytotoxicity of Al_2_O_3_ NPs was evaluated by CCK-8 assay. HBE cells were exposed to Al_2_O_3_ NPs at different doses (12.5-1000 μg/ml) every 24 h up to 7 days. 100 μg/ml or higher doses showed significant toxicity to HBE cells 24 h after treatment (Fig. 4a). To further verify that Al_2_O_3_ NP exposure caused apoptosis, cells was assessed by flow cytometric analysis of annexin V-FITC and PI double staining. After 12 h treatment, significant enhancement of apoptosis (2.24 ± 0.17 %) was observed only in 500 μg/mL Al_2_O_3_ NPs HBE cells (Additional file 1: Figure S1A). Further, apoptosis occurred 8.98 ± 0.39 % and 16.77 ± 0.57 % in HBE cells treated with 100 or 500 μg/mL Al_2_O_3_ NPs for 24 h, respectively, but significant increase of cellular necrosis was not observed at either treatment concentrations (Fig. 4b). To determine pathways induced by Al_2_O_3_ NP exposure, caspase-3, 8, and -9 activities were evaluated. Increased activities of caspase-3 and -9 were shown in Al_2_O_3_ NPs treated groups, suggesting HBE cell apoptosis is initiated by the intrinsic pathway (Fig. 4c). Similar results were obtained from lung tissues harvested from mice treated with Al_2_O_3_ NPs (Fig. 4c).

**Fig. 5:**
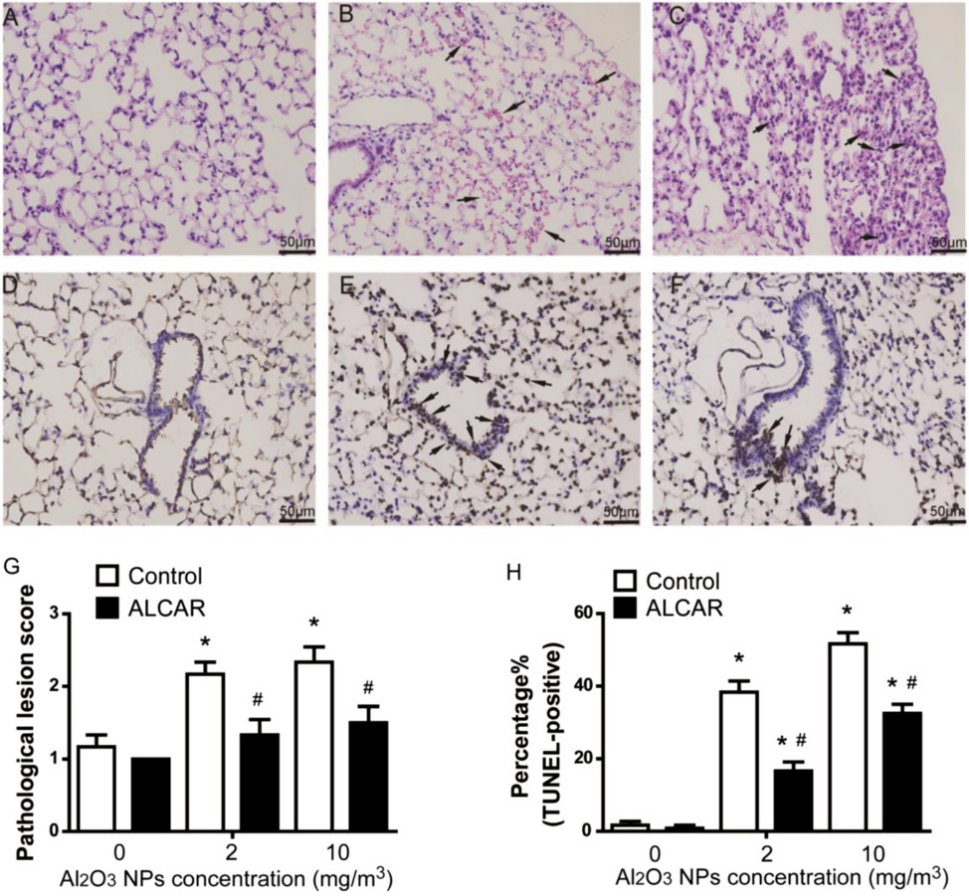
HE and TUNEL staining of lung tissues harvested from mice exposed to Al_2_O_3_ NPs. **a** HE staining of control mice lung tissue **b**) arrows showed pneumorrhagia in lung tissue from 2 mg/m^3^ Al_2_O_3_ NP exposure **c**) arrows demonstrated lymphocytes infiltration of lung tissue due to exposure to 10 mg/m^3^ Al_2_O_3_ NPs **d**) few apoptosis cells were observed in control mice lung tissue by TUNEL staining **e**) massive apoptosis in bronchial epithelia were induced by 10 mg/m^3^ Al_2_O_3_ NP exposure as shown by arrows **f**) ALCAR supplement reduced apoptosis of bronchial epithelia in 10 mg/m^3^ Al_2_O_3_ NP exposed mice lung tissue. The arrows show apoptotic epithelial cells. **g** The pathological lesion score of mice lung tissues **h**) Proportion of TUNEL-positive bronchial epithelia within each group. ^*^ *P* < 0.05, compared with untreated control. ^#^ *P* < 0.05, compared with corresponding control

H&E and TUNEL staining was performed to detect pathology and programmed cell death *in situ*. Inflammation and red blood cell located in pulmonary mesenchyme were observed in both low and high dose exposed mice lung tissue. The typical pathological alterations were showed in Fig. 5. Figure 5a showed a normal structure of lung tissue. As shown in Fig. 5b, pneumorrhagia characterized by interstitial red blood cells distribution was seen in lung tissue obtained from mice treated with low dose Al_2_O_3_ NPs. Massive lymphocytes infiltration, especially the subpleural area, were observed in high dose Al_2_O_3_ NPs treated mice lung tissue (Fig. 5c). The pathological lesions scores were showed in Fig. 5g. Compared with control group, the pathological lesion score increased significantly in both nanoparticle treated lung tissues (*P* < 0.05), However, high dose exposure did not enhance the pathological lesions in lung tissue significantly when compared with low Al_2_O_3_ NPs treatment. For both treatment groups, pathological scores suggested mild to moderate alveolitis in lung tissues of mice, further, following ALCAR rescue, the alveolitis were attenuated to mild degree. ALCAR ameliorated the damages against Al_2_O_3_ NPs significantly (*P* < 0.05), compared with corresponding Al_2_O_3_ NPs treated groups.

TUNEL-positive cells were not observed in the bronchial epithelia in untreated mice lung (Fig. 5d). Treatment with 10 mg/m^3^ Al_2_O_3_ NPs resulted in degeneration of lung cells and the massive bronchial epithelial cells had significant apoptosis (Fig. 5e) Ameliorated apoptosis was observed in bronchial epithelia of mice exposed to high dose Al_2_O_3_ NPs following ALCAR rescue (Fig. 5f). The proportion of TUNEL-positive bronchial epithelia were showed in Fig. 5h. For both low and high dose Al_2_O_3_ NPs treatment, ALCAR could partially rescue apoptosis of bronchial epithelia to control level.

### Dysfunction of mitochondria induced by Al_2_O_3_ NPs

**Fig. 6:**
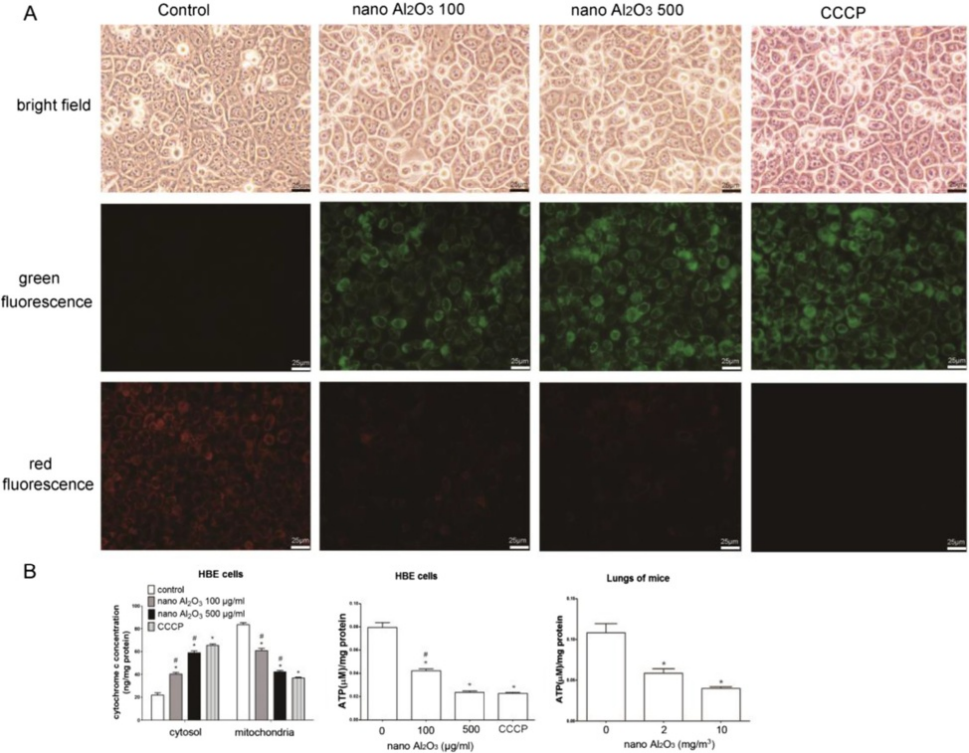
Mitochondrial dysfunction is observed in both HBE cells and in lung tissues treated with Al_2_O_3_ NPs. **a** JC-1 staining showed alteration of mitochondrial membrane potential. The left panel shows cells from control group indicating that only red fluorescence was observed. After treatment with 100 or 500 μg/ml Al_2_O_3_ NPs, increased green fluorescence and decreased red fluorescence were observed, which suggested collapse of mitochondrial membrane potential. CCCP was employed as positive control and demonstrated strong damages to mitochondrial membrane potential **b**) Cytochrome *c* is released from mitochondria, therefore, cytosol cytochrome *c* concentration was enhanced in HBE cells. Meanwhile ATP synthesis decreased in both HBE cells and lung tissues exposed to Al_2_O_3_ NPs. ^*^ *P* < 0.05, compared with untreated control, ^#^ *P* < 0.05, compared with positive control (CCCP)

The mitochondria-mediated apoptosis pathway is characterized by destruction of mitochondrial membrane potential and subsequent release of cytochrome *c* into the cytoplasm [6], accompanied by decreased ATP synthesis. The fluorescent probe JC-1 staining showed red fluorescence in normal cells, indicating intact mitochondrial membrane potential, while cells treated with 100 and 500 μg/ml Al_2_O_3_ NPs were stained green after 12 or 24 h treatment, indicating clear damage to the mitochondrial membrane potential as shown in Additional file 1: Figure S1B and Fig. 6a. Additionally, cytoplasmic cytochrome *c* significantly increased in cells treated with Al_2_O_3_ NPs (Fig. 6b), however, this was lower than the positive control (CCCP).

ATP levels were detected by luminometer after 24 h exposure. In both HBE cells and lung tissues from mice treated with Al_2_O_3_ NP, ATP decreased compared to the control (Fig. 6b). Collectively, our results suggest Al_2_O_3_ NPs exposure is cytotoxic.

### Al_2_O_3_ NPs induce oxidative stress in both HBE cells and mice lung tissues

**Fig. 7:**
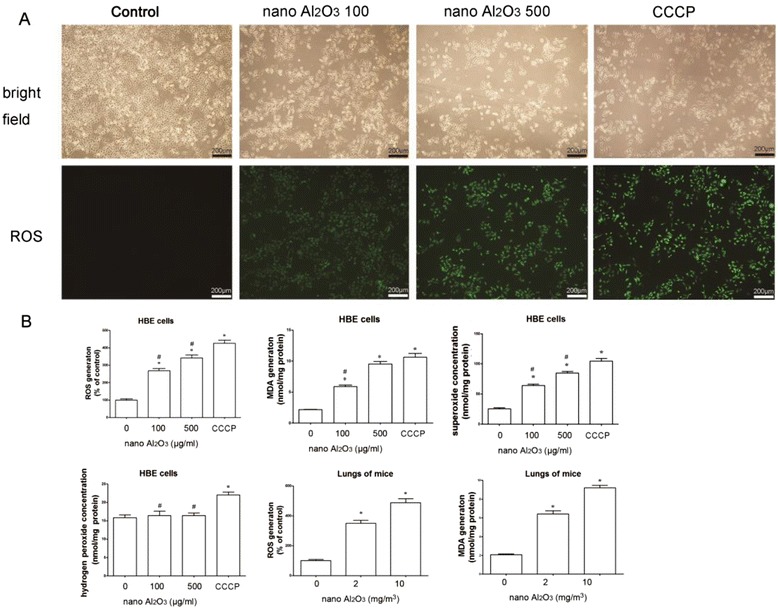
Oxidative stress and lipid peroxidation induced by Al2O3 NP exposure. **a** ROS is induced by Al_2_O_3_ NP exposure in HBE cells with increased green fluorescence. CCCP was employed as positive control. **b** ROS, O_2_^.-^ and lipid peroxidation production MDA are increased in HBE cells and mouse lungs following Al_2_O_3_ NP exposure. The H_2_O_2_ level is not affected by Al_2_O_3_ NPs exposure in HBE cells. ^*^ *P* < 0.05, compared with untreated control, ^#^ *P* < 0.05, compared with positive control (CCCP)

To assess the importance of oxidative stress in Al_2_O_3_ NP cytotoxicity, ROS, H_2_O_2_, O_2_^.-^ and MDA levels were evaluated in HBE cells 12 or 24 h after treatment. Correspondingly, ROS and MDA levels were evaluated in mice lung tissues. ROS generation is a main cause of mitochondria-dependent apoptosis. We used DCFH-DA as a reporter of ROS generation, and as shown in Fig. 7a, level of ROS increased in cells treated with Al_2_O_3_ NPs for 24 h, indicated by higher intensity of fluorescence. As shown in Additional file 1: Figure S1C and Fig. 7b, Al_2_O_3_ NP treatment significantly increased ROS and O_2_^.-^ levels, but not H_2_O_2_ levels, in HBE cells. Comparable results were observed in lungs obtained from mice treated with Al_2_O_3_ NPs. Similarly, MDA, the end product of lipid peroxidation, also increased as shown in Fig. 7b and Additional file 1: Figure S1C. For all of these oxidative stress-involved assays, the positive control (CCCP) demonstrated stronger stimulus to HBE cells than Al_2_O_3_ NPs.

### Metabolic analysis showed up- and down- regulated metabolites of HBE cells

According to PCA analysis, significant differences were observed between control HBE cells and HBE cells treated with either 100 μg/ml or 500 μg/ml Al_2_O_3_ NPs (Additional file 1: Figure S2). We used MetaboAnalyst to identify key metabolism alterations between untreated and treated HBE cells. After Bonferroni correction (*p* cut-off < 0.00001), we found 9 down-regulated and 11 up-regulated small molecules in treated samples in a dose-dependent manner (Table 2). Among differentially regulated metabolites, acetyl-L-carnitine (ALCAR) (*p* = 3.64E^−09^), 4-Methoxyphenylacetic acid (*p* = 7.61E^−07^) and thymine (*p* = 2.99E^−07^) were the lowest in *P* value. Because ALCAR is important for mitochondrial function, it could be a candidate supplement to rescue the adverse effects of Al_2_O_3_ NPs on HBE cells.

**Table 2:** Metabolomics analysis of Al_2_O_3_ NPs exposed HBE cell lysates

Peak	Metabolic pathway	HMDB	Regulation
3-Phenylbutyric Acid	Taurine and Hypotaurine Metabolism	HMDB01955	down
Melatonin	Tryptophan Metabolism	HMDB01389	down
cis-Aconitic acid	Citric Acid Cycle	HMDB00072	down
3-Methylhistidine	Histidine Metabolism	HMDB00479	down
Acetyl-L-carnitine	Beta Oxidation of Very Long Chain Fatty Acids	HMDB00201	down
4-Methoxyphenylacetic acid	Tyrosine Metabolism	HMDB02072	down
N-Acetyl-L-methionine	Betaine Metabolism	HMDB11745	down
N-Formyl-L-methionine	Betaine Metabolism	HMDB01015	down
Pyridoxal 5′-phosphate	Vitamin B6 Metabolism	HMDB01491	down
L-Serine	Homocysteine Degradation	HMDB00187	up
Adenine	Phytanic Acid Peroxisomal Oxidation	HMDB00034	up
Succinic acid	Aspartate Metabolism	HMDB00254	up
D-Glutamic acid	Glutamate Metabolism	HMDB03339	up
Thymine	Pyrimidine Metabolism	HMDB00262	up
Ethylmalonic acid	Mefanamic Acid Pathway	HMDB00622	up
Ureidopropionic acid	Beta-Alanine Metabolism	HMDB00026	up
(-)Matairesinol	Phenylacetate Metabolism	HMDB35698	up
Taurocholic Acid	Bile Acid Biosynthesis	HMDB00036	up
Docosahexaenoic acid	Mefanamic Acid Pathway	HMDB02183	up
Pyridoxine	Vitamin B6 Metabolism	HMDB00239	up

### Protective effects of ALCAR to HBE cells against Al_2_O_3_ NPs induced mitochondria dysfunction and oxidative stress

**Fig. 8:**
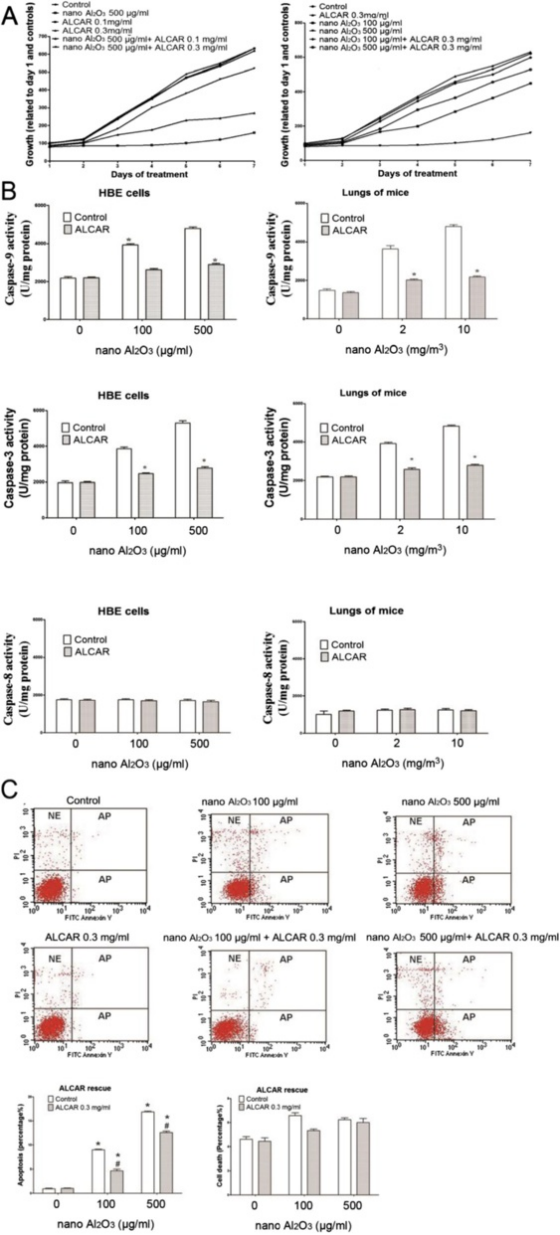
The ameliorative effects of ALCAR supplementation against cell viability lost and apoptosis. **a** 0.3 mg/ml ALCAR co-treatment effectively increased cell viability. **b** 0.3 mg/ml ALCAR co-treatment with Al_2_O_3_ NPs ameliorated activities of caspase-3 and -9 completely or partially to control level in HBE cells by **c**) 0.3 mg/ml ALCAR co-treatment partially protected HBE cells from apoptosis induced by Al_2_O_3_ NPs. ^*^ *P* < 0.05, compared with untreated control, ^#^ *P* < 0.05, compared with corresponding control within each treatment group

**Fig. 9:**
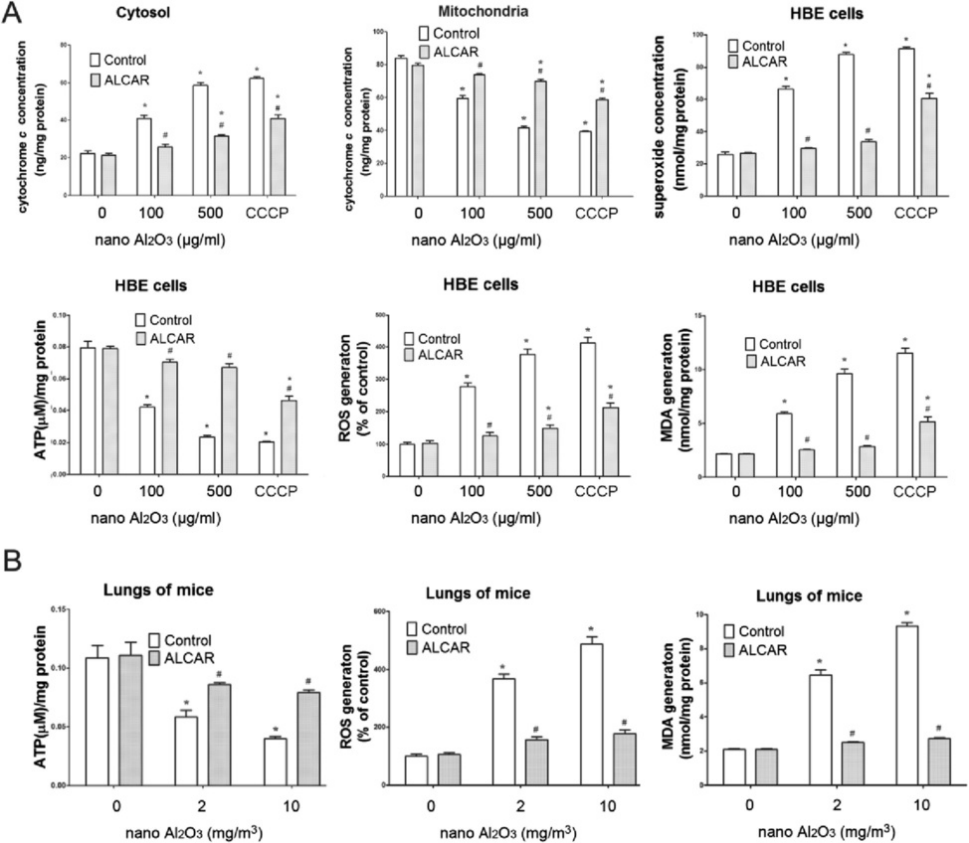
Oxidative stress and lipid peroxidation are reduced after ALCAR rescue. **a** Oxidative stress and lipid peroxidation are reduced by ALCAR supplementation in HBE cells and **b**) lung tissues from mice treated with Al_2_O_3_ NPs. ^*^ *P* < 0.05, compared with untreated control, ^#^ *P* < 0.05, compared with corresponding control within each treatment group

To test whether ALCAR could reduce cell toxicity due to Al_2_O_3_ NPs, toxicity was evaluated in cells treated with 0.1 or 0.3 mg/ml ALCAR at treatment with 500 μg/ml Al_2_O_3_ NPs. As shown in Fig. 8a, 0.3 mg/ml ALCAR treatment significantly reduced the cell viability loss in both 100 and 500 μg/ml Al_2_O_3_ NP treated cells, and was used as a therapeutic dose for the remaining experiments. Compared to untreated cells and lungs, co-treatment with ALCAR attenuated caspase-3 and -9 activities as shown in Fig. 8b. Caspase-8 activity was not affected by ALCAR treatment (Fig. 8b). To confirm ALCAR treatment reduced apoptosis, treated cells were evaluated by annexin-V and PI staining. Similarly, 0.3 mg/ml ALCAR treatment reduced annexin-V staining significantly (Fig. 8c). ALCAR treatment also reduced production of intracellular ROS, O_2_^.-^ and MDA in HBE cells and lungs harvested from mice treated with Al_2_O_3_ NPs as shown in Fig. 9a and b. ALCAR could only partially rescue HBE cells against CCCP induced oxidative stress (Fig. 9a). ALCAR also demonstrated protective effects to mice bronchia epithelia from apoptosis (Fig. 5f).

**Fig. 10:**
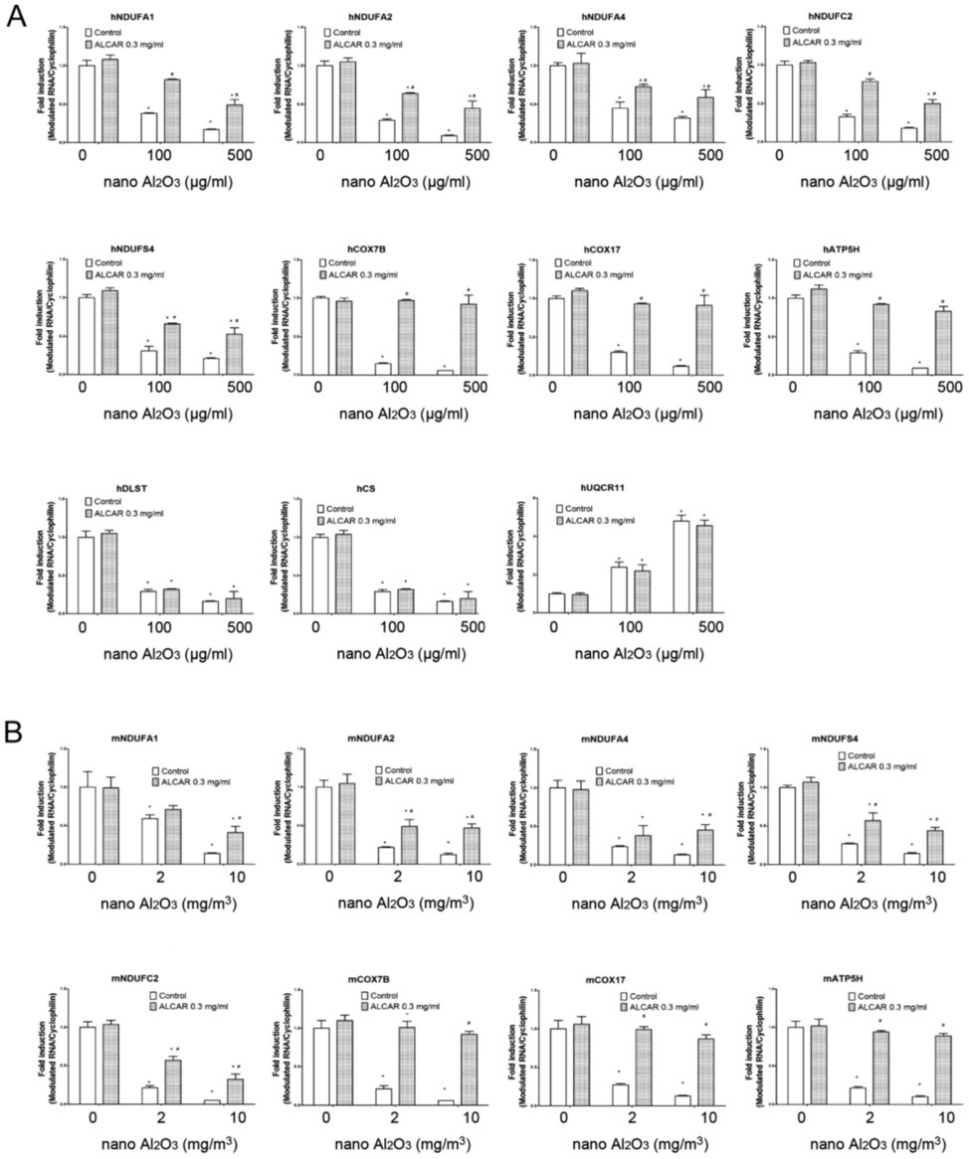
Gene expression levels after ALCAR rescue. **a** Expression levels of genes located in complex I, IV and V are partially or completely normalized after ALCAR rescue in HBE cells and in **b**) lung tissues from mice exposed to Al_2_O_3_ NPs

Gene expression levels in HBE cells and mouse lung tissues after ALCAR treatment were evaluated by qRT-PCR. Altered expression of genes located in mitochondria complex IV and V, COX7B, COX17 and ATP5H, was comparable to control cells following ALCAR treatment. Expression of genes in complex I, including NDUFA1, NDUFA2, NDUFA4, NDUFC2 and NDUFS4 were partially normalized following ALCAR treatment. UQCR11, DLST, and CS expression was unresponsive to ALCAR treatment (Fig. 10).

## Discussion

Using high-throughput mRNA sequencing and computational approaches, we showed that Al_2_O_3_ NPs cause changes in gene expression in HBE cells. Among the differentially expressed genes, mitochondrial genes were among the most significantly affected category. Further, cell and animal based functional assays confirmed that Al_2_O_3_ NPs caused mitochondrial dysfunction and oxidative stress in HBE cells and mouse lung tissue. Metabolomics analysis showed downregulation of ALCAR in HBE lysate in a dose-dependent manner following Al_2_O_3_ NP treatments. Co-treatment of cells with ALCAR and Al_2_O_3_ NPs prevented mitochondrial dysfunction both in vivo and in vitro.

Inhalation is one of the major exposure routes for nano-scale materials, including engineered nanoparticles and ambient particulate matter (PM), which have effects on the respiratory system [28]. Various nanoparticles are reported to produce pulmonary lesions. For example, respiratory administration of single-walled carbon nanotubes (SWCNTs) increased collagen and alveolar wall thickness [29]. Well dispersed nickel-oxide nanoparticles increase inflammation and cytotoxicity in C57BL/6 J mice lung tissues [30]. Transcriptomic and proteomic approaches implicate that inflammation and oxidative stress are involved in pulmonary toxicity caused by silica nanoparticles [31]. Chronic exposure to ambient PM can cause lung remodeling, with particle deposition on terminal bronchioles and first-generation respiratory bronchioles [32]. Though aluminum is considered as a neural toxin, lung tissue could be the primary organ affected by inhaled nano-scale Al_2_O_3_ particles from either engineered or ambient sources. According to the Chinese Health standard for dusts of aluminum, aluminum oxide, and aluminum alloys in the air of workplace (GB11726-89), the maximum allowable concentration for Al_2_O_3_ powder in air is 6 mg/m^3^. Among these suspended particles, the health effects of nano-scaled Al_2_O_3_ particles should be intensively considered due to its longer suspension and lower deposition in respiratory system than other large particles. In the present study, the exposure doses of Al_2_O_3_ NPs to mice were set to 2 or 10 mg/m^3^ to evaluate the potential pulmonary damages.

Mitochondrial function was used as an indicator to evaluate the biological effects of various nanomaterials [33]. Copper oxide nanoparticles induce apoptosis in human hepato-carcinoma (HEPG2) cells through the intrinsic apoptosis pathway, which is accompanied by increased reactive oxygen species generation and oxidative stress and the collapse of mitochondria membrane potential [34]. Nymark et al [33] combined transcriptome and microRNA analysis to identify mitochondrial dysfunctions induced by multi-walled carbon nanotubes and 26 genes with known mitochondrial function were identified. Additionally, GO analysis showed that the most significantly enriched biological process among the 26 differentially expressed genes was gluconeogenesis. Our studies suggest mRNA profiling analysis is a reasonable strategy to study the Al_2_O_3_ NP toxicity on HBE cells. A total of 27 genes associated with the mitochondria correlated to Al_2_O_3_ NP treatment. In contrast to Nymark’s results, our array data suggested that oxidative phosphorylation was the most significantly affected biological process in treated cells. Gene expression levels were confirmed by qRT-PCR in HBE cells and mouse lung tissues exposed to Al_2_O_3_ NPs, further implicating altered mitochondrial function.

Mitochondria-mediated apoptosis is initiated by mitochondrial outer membrane permeabilization and release of cytochrome *c* into the cytoplasm [6]. Cytochrome *c* binds to apoptosis protease activating factor-1 (Apaf-1), which leads to activation of caspase-9 and -3, and is essential for DNA damage and apoptosis [35]. To determine whether differential expression of mitochondrial genes may lead to apoptosis, we monitored cell viability and mitochondrial function in cells and tissues exposed to Al_2_O_3_ NPs. Decreased mitochondrial membrane potential, increased caspase-9 and -3, and increased apoptosis were observed in cells and lung tissues exposed to Al_2_O_3_ NPs, suggesting Al_2_O_3_ NPs causes cytotoxic effects in the pulmonary tract.

Mitochondria are the primary source of cellular ROS, produced through oxidative phosphorylation, especially at levels of complexes I and III of the respiratory chain [36]. Excessive ROS production can lead to mitochondrial oxidative damage, which can lead to cell apoptosis. O_2_^.-^ is an indicator of mitochondrial ROS production, and is generated by the addition of one electron to O_2_. Most intracellular ROS are derived from superoxide O_2_^.-^ and elicit various pathological events, including DNA damage and apoptosis [37–39]. Due to the small size and large surface area of nanoparticles, ROS are usually produced when cells are exposed to nanoparticles. As a result, oxidative stress and lipid peroxidation have been hypothesized to play an important role in nanoparticle toxicity [34, 40]. In the present study, alteration of mitochondrial membrane potential, ROS and lipid peroxidation were significantly higher in HBE cells exposed to Al_2_O_3_ NPs after 12 or 24 h treatment, while prominent enhancement of apoptosis and gene modulation were observed 24 h after treatment. Therefore, the mitochondria-involved dysfunction could account for Al_2_O_3_ NPs induced gene modulations and subsequent apoptosis. Significantly, the antioxidant ALCAR reduced cell apoptosis effectively, suggesting that mitochondria associated oxidative stress is involved in Al_2_O_3_ NP cytotoxicity.

The carnitine: acylcarnitine translocase (CACTL) located in the mitochondrial inner membrane facilitates cytosolic ALCAR entry into the mitochondria [41]. Inside the mitochondrial matrix, carnitine palmityltransferase II releases carnitine from ALCAR. Carnitine is then transported back across the membrane by carnitine-acylcarnitine translocase [42]. Therefore, ALCAR is considered a specific intramitochondrial acetyl-donor and may be involved in restoring mitochondrial function [43]. ALCAR and its derivative L-carnitine supplementation have wide clinical applications to restore neurologic disorders [44] or improve liver function [42]. Our studies using metabolomics suggest ALCAR levels decrease dramatically due to Al_2_O_3_ NP exposure. Because ALCAR is reported to improve mitochondrial function and restore mitochondrial gene expression, [45] we supplemented HBE cells and mice with ALCAR during exposure to Al_2_O_3_ NPs. ALCAR treatment significantly increased cell viability and decreased apoptosis, corresponding to decreased caspase activity and cytochrome *c* release. Additionally, ALCAR reduced oxidative stress and lipid peroxidation due to Al_2_O_3_ NP exposure. ALCAR supplementation fully or partially rescued altered gene expression of mitochondrial-associated proteins due to Al_2_O_3_ NP exposure. Consistent with our studies, treating rats with ALCAR rescued the function of complexes I, IV and V in brain mitochondria [46]. ALCAR additionally reduced ROS production and ATP depletion in an in vitro cell model [26]. We observed that ALCAR could partially restore oxidative stress and lipid peroxidation induced by a powerful mitochondrial uncoupling agent CCCP, therefore demonstrating strong anti-oxidative activities in the present study.

NADH dehydrogenase (complex I) and cytochrome *c* oxidase (complex IV) are highly susceptible to oxidative damage [47, 48]. Our data indicate that genes involved in complex I, IV, and V were significantly down-regulated in both Al_2_O_3_ NP-treated HBE cells and in lung tissues harvested from mice exposed to Al_2_O_3_ NPs, suggesting that loss of mitochondrial function may be due to compromised NADH-linked respiration and complex I-driven electron transport. Furthermore, our data suggests that ALCAR may be useful in maintaining ATP levels under specific pathologic conditions [21]. ALCAR can partially restore the gene expression level in complex I and completely restore gene expression level in complex IV and V, consistent with previous reports that ALCAR protects key mitochondrial enzymes (complex I and IV) from oxidative damage.

In the present study, ALCAR rescued HBE cells against Al_2_O_3_ NPs induced apoptosis, which is attributed to the anti-oxidative activities and gene restoration. Further, ALCAR could be a specific supplement according to our metabolomics assays because it was the most significantly down-regulated small molecule metabolite following Al_2_O_3_ NPs exposure. However, studies on the involved metabolic pathways and underlying mechanisms are further required.

## Conclusions

In summary, we present a approach based on transcriptomics coupled with metabolomics methods, to reveal key genes involved in response to engineered Al_2_O_3_ NPs. These nano-scale Al_2_O_3_ particles are abundant in either occupational environments or as components of particulate matters in the ambient environment. RNA microarray revealed that down-regulated genes were mainly associated with mitochondrial complexes I and IV and oxidative stress. Treatment with the antioxidant ALCAR which is screened according to the metabolomics analysis was tested in our metabolomics study, and restored mitochondrial function through both in vivo and in vitro studies. We suggest that our “omics” technologies can be used to identify specific pathways involved in toxicities due to Al_2_O_3_ NPs and provides insight into identifying potential treatment solutions. Our study is the first to demonstrate the use of ALCAR to restore mitochondrial function and protect cell toxicity from Al_2_O_3_ NP exposure in vivo and in vitro.

## Methods

### Nanomaterials and animals

Al_2_O_3_ NPs were purchased from Plasmachem GmbH, Germany (>99.8 % purity). The particle size and zeta potential of Al_2_O_3_ NPs in PBS suspension was 64.17 nm and 37.1 mV, which was analyzed by a zetasizer (nano-zs90, Malvern Instruments, UK) (Additional file 1: Figure S3).

Thirty-six male and 36 female ICR mice (20–22 g) were purchased from Shanghai SLRC Laboratory Animal Co. Ltd., China.

### Cell culture and RNA extraction

The human bronchial epithelial cell line (HBE) (American Type Culture Collection) was maintained in Dulbecco’s modified Eagle’s medium (DMEM) at 37 °C in 5 % CO_2_. The culture medium was supplemented with 10 % (v/v) fetal bovine serum (FBS), penicillin (100 U/ml), and streptomycin (100 μg/ml). Cells were seeded in 10 cm culture dishes and exposed to 100 μg/ml Al_2_O_3_ NPs with three biological replicates. At 24 h post treatment, complete medium was removed and adherent cells were collected. Total RNA was extracted using the TRIZOL reagent (Invitrogen, U.S.) according to the manufacturer’s instructions.

### RNA microarray and gene expression analysis

An Agilent Array platform (Agilent Technologies, Santa Clara, CA, USA) was employed for microarray analysis. The labeled cRNAs were hybridized onto a Human LncRNA Array v3.0 (8 × 60 K; arraystar) chip designed for 26 109 coding genes. The arrays were scanned using an Agilent G2505C scanner and the fluorescence intensity was analyzed with Agilent Feature Extraction software (version 11.0.1.1). Quantile normalization and subsequent data processing were performed using the GeneSpring Gx v12.0 software package (Agilent Technologies). An absolute fold change of 2 or more and 0.05 adjusted P-value were set as cutoff to evaluate the significance of gene expression differences of raw data.

### Functional group analysis

DAVID 6.7 (Database for Annotation, Visualization, and Integrated Discovery), a functional annotation tool, was used to analyze differentially expressed genes. The DAVID functional annotation cluster tool provides three structured networks of defined terms that describe the attributes of gene products. The P-value was set to 0.1 to denote the significance of GO enrichment in the differentially expressed mRNA list. Fold enrichment (-lg (*P*-value)) was used to denote the enrichment of a particular GO term. Pathway analysis for differentially expressed mRNAs was performed on the KEGG database. This analysis determines the biological pathways for which a significant enrichment of differentially expressed mRNAs exist (*P*-value was set as 0.05).

### Metabolomics analysis of Al_2_O_3_ NPs treated HBE cells

GC/TOFMS analysis was performed using an Agilent 7890 gas chromatography system coupled with a Pegasus 4D time-of-flight mass spectrometer. HBE cells were exposed to Al_2_O_3_ NPs (0, 100, and 500 μg/ml) for 24 h with 9 biological replicates. Then the cell lysate sample were prepared and analyzed as described [16].

### Animal treatment

Mice were maintained and used according to the guidelines of the Committee on Animal Use and Care of Southeast University. The dynamic inhalation exposure chambers were outfitted with an aerosol generator (Beijing HuiRongHe Technology Co., Ltd, China). . Mice were divided into six groups (with six male and six female mice in each group): control, mice treated with 100 mg/kg ALCAR, mice treated with low and high doses of Al_2_O_3_ NPs, mice treated with a low dose of Al_2_O_3_ NPs + 100 mg/kg ALCAR, and mice treated with a high dose of Al_2_O_3_ NPs + 100 mg/kg ALCAR. Mice were housed six per polycarbonate cage on corncob bedding with ad libitum access to food and water. Exposure was carried out in three whole-body inhalation chambers; two chambers received low or high dose of Al_2_O_3_ NPs and the control chamber received HEPA-filtered clean air at the same flow rates as the experimental groups. Six males and six females were exposed in each chamber for 8 h per day for 7 consecutive days. Light cycles were set on a 12/12 h light/dark cycle. Al_2_O_3_ NPs in the chamber were generated by the aerosol generator and mean concentrations of Al_2_O_3_ NPs were 2 mg/m^3^ and 10 mg/m^3^ for low and high dose treatments, respectively. The concentration of Al_2_O3 NPs were monitored by an extensive air quality monitoring equipment (CEL-712 Microdust Pro, CASELLA, UK). 100 mg/kg ALCAR was orally administrated by gavage. The temperature, air flow rate and relative humidity in the chambers was set to 22.5 °C, 40 L/min, and 50 ± 10 %.

### Histopathological analysis of mice lung tissue

Mice were euthanized under ether anesthesia 1 h after the end of dynamic Al_2_O_3_ NP exposure on the 7^th^ day. All the mice were decaptitated on an iced table. Lung tissue was divied for three parts. One piece was immediately prepared for capasse activity, ATP level, ROS and MDA analysis. The second piece was stored in liquid nitrogen, while another piece was preserved in 4 % paraformaldehyde (PFA) for 24 h at 4 °C, embedded in paraffin, serially sectioned (5 μm) and mounted on silane-covered slides. After dewaxing, the sections selected from each mouse were stained with hematoxylin and eosin (HE) and evaluated under a light microscope (400×) to examine the histology of the lung tissues. The severity of pathological lesions was scored according to Szapiel’s method (Additional file 1: Table S1) [49].

Apoptotic cells in lung tissues were evaluated through Terminal-deoxynucleoitidyl transferase mediated nick end labeling (TUNEL) staining by a Roche In Situ Cell Death Detection Kit (Roche, U.S.) according to the suggested protocols. The proportion of TUNEL-positive cells of bronchial epithelia were estimated by an experienced histologists blinded to treatment conditions. Five non-overlapping bronchial tubes of each section were counted in high-power fields (HPFS, ×400 magnification) and analyzed. The bronchial tubes had a maximum of positive cells were selected.

### Cell viability

Cellular viability was evaluated by a CCK-8 proliferation assay using a Cell Counting Kit-8 (Nanjing Jiancheng Bioengineering Institute, China). HBE cells were plated at a density of 1 × 10^4^ per well in a 96-well plate and treated with 0, 12.5, 25, 50, 100, 250, 500, 1 000 μg/ml (corresponding to 0, 3.91, 7.81, 15.62, 31.25, 78.13, 156.25, 312.5 μg/cm^2^) Al_2_O_3_ NPs with eight biological replicates for each concentration. 10 μL of CCK-8 was added to each well, cells were incubated for 4 h at 37 °C, and the absorbance was determined at 450 nm. Cell viability affected by Al_2_O_3_ NPs were monitored every 24 h for up to 7 days.

### Cell apoptosis analysis

Apoptosis was assessed by flow cytometry using a KeyGEN Annexin V-FITC Apoptosis Detection Kit (KeyGEN BioTECH, China) according to the manufacturer’s instructions. Briefly, after exposure to 100 or 500 μg/ml Al_2_O_3_ NPs for 12 or 24 h, HBE cells were harvested and incubated with 5 μl FITC-conjugated annexin V and 5 μl PI for 15 min at room temperature in the dark. The samples were analyzed by a FACS Calibur Flow Cytometer (BD Biosciences, USA).

### Assessment of caspase-3, caspase-8, and caspase-9 activities

Caspase activity assay kits (Beyotime Institute of Biotechnology, China) were used to evaluate caspase-3, 8, and 9 activities in HBE cell lysates and lung tissues according to the manufacturer’s instructions. Briefly, 0.05 g lung tissues were homogenized with 500 μl lysis buffer on ice. cells were lysed with lysis buffer. Protein content was measured by the Bradford method. Ac-DEVD-pNA, Ac-IETD-*p*NA, Ac-LEHD-*p*NA were substrate peptides of caspase-3, 8, and 9 respectively, and incubated with Al_2_O_3_ NPs-treated cell or lung tissue lysate for 2 h at 37 °C. The release of p-nitroanilide (pNA) from substrates was measured at 405 nm by a microplate reader (Molecular Devices, U.S.).

### Mitochondria functions analysis

ATP levels were measured using a luciferase ATP assay kit (Beyotime, China). Briefly, 200 μL of lysis buffer was added to cells treated or untreated with Al_2_O_3_ NPs. Cells were collected and centrifuged at 12 000 rpm for 5 min at 4 °C. 0.05 g lung tissue were homogenized with 250 μl lysis buffer, then centrifuged at 12,000 rpm for 5 min at 4 °C. The luminescence of the supernatant was assayed by a luminometer (Berthold Detection System, Pforzheim, Germany). The cationic dye JC-1 was used to detect the mitochondrial membrane potential, and HBE cells exposed to Al_2_O_3_ NPs 12 or 24 h were evaluated under a fluorescence microscope (Olympus, Japan) to examine green and red fluorescence.

To determine the release of cytochrome *c*, mitochondrial and cytosolic proteins were isolated by a Mitochondria/Cytosol Fractionation Kit (Beyotime, China) according to the manufacturer’s instructions. The protein concentrations in cytosol and mitochondria samples were measured using the Bradford method. The levels of cytochrome *c* were estimated according to the ELISA kit procedures (R&D Systems, U.S.). For all of these mitochondrial-involved assays, CCCP was employed as positive control.

### Measurements of reactive oxygen species (ROS), superoxide (O_2_^.-^), hydrogen peroxide (H_2_O_2_) and malondialdehyde (MDA) levels

After 12 or 24 h exposure to 100 or 500 μg/ml Al2O3 NPs, HBE cells were washed with PBS. 0.05 g fresh mice lung were homogenized by with 1 mL PBS, and centrifuged at 1,600 g for 10 min at 4 °C. Then DCFH-DA probe was added (Beyotime, China) with a final concentration of 10 μmol/L to determine the content of intracellular ROS. The fluorescence intensity was measured after 30 min incubation at 37 °C under a fluorescence microscope (Olympus, Japan), and quantified by a fluorescence spectrophotometer. O_2_^.-^ levels in the cell lysate were determined by WST-1 assay. The concentration of formazan, the reduced product of NBT was measured at 560 nm. H_2_O_2_ concentration in cells was determined by optical density (OD) at 560 nm, indicative of H_2_O_2_ oxidization of trivalent ferric and xylenol orange. The concentrations of MDA in cellular and lung tissue lysate were assessed by measuring thiobarbituric-acid (TBA) reacting substances at 532 nm. The level of MDA was expressed as nmol MDA per milligram protein. Protein content was measured by Bradford method. For all of these oxidative stress and lipid peroxidation related assays, CCCP was employed as positive control.

### ALCAR rescue to Al_2_O_3_ NPs induced mitochondrial dysfunction

Cell viability of HBE cells treated with PBS, 0.1, 0.3 mg/ml ALCAR, 500 μg/ml Al_2_O_3_ NPs, 0.1 mg/ml ALCAR + 500 μg/ml Al_2_O_3_ NPs, or 0.3 mg/ml ALCAR + 500 μg/ml Al_2_O_3_ NPs was assessed to determine an appropriate therapeutic dose of ALCAR.

To validate the protective effects of ALCAR against mitochondrial dysfunction, HBE cells were treated with PBS, 100, 500 μg/ml Al_2_O_3_ NPs, 0.3 mg/ml ALCAR, 0.3 mg/ml ALCAR+ 100 μg/ml Al_2_O_3_ NPs or 0.3 mg/ml ALCAR+ 500 μg/ml Al_2_O_3_ NPs for 24 h. Then mitochondrial membrane potential, ROS, ATP, MDA levels, caspase-3, 8, and 9 activities, and O_2_^.-^ levels in HBE cells were determined.

### RNA isolation and quantitative real-time PCR assay

HBE cells were seeded in 6-well plates at a density of about 1 × 10^6^ cells per well, and exposed to 50, 100, 250, or 500 μg/ml Al_2_O_3_ NPs or control medium for 24 h, then cells were trypsinized and collected. The mice lung tissue stored in liquid nitrogen were homogenized in ice-cold 50 mM Tris–HCl buffer (pH 7.55).

Total RNA of HBE cells and lung tissues were extracted using a GenElute^TM^ Mammalian Total RNA Miniprep Kit (Sigma, U.S.) according to the manufacturer’s protocol, and the concentration of total RNA was determined by measuring the absorbance at 260 nm using a Nanodrop 2000c spectrophotometer (Thermo Scientific, U.S.). cDNA synthesis for coding genes was performed with 1ug of total RNA according to the manufacturer’s instruction (Takara, Japan).

The mRNA levels for modulated genes were determined by reverse transcription of total RNA followed by quantitative real-time PCR analysis (qRT-PCR) on a Quant Studio 6 Flex system (Applied Biosystems, Life Technologies, U.S.) using SYBR PCR Master Mix reagent kits (Takara, Japan) following the manufacturer’s protocol. Primers were designed for the modulated genes screened by RNA microarray, and are provided in Additional file 1: Table S2 and S3. All experiments were performed in triplicate. The mRNA levels provided were normalized to cyclophilin A.

### Data analysis

Values of cell viability, apoptosis, and mitochondrial dysfunction assays are expressed as mean ± standard error of the mean (SE). Statistically significant differences were determined by one-way ANOVA, followed by Dunnett’s multiple comparison tests. Kruskal-Wallis test was used to analyze the ranked data of pulmonary lesion scores. The method of 2 ^–ΔΔCt^ was used to analyze the results of RT-PCR in all experiments. Statistical analysis was performed by SPSS12.0 and the significance was set at *P* < 0.05.

## Additional file

Additional file 1:
**Supplementary documents Figure S1-3 with figure legend and Tables S1-3.** (PDF 793 kb)

## Abbreviations

Al_2_O_3_: aluminum oxide
ALCAR: acetyl-L-carnitine
Apaf-1: apoptosis protease activating factor-1
CACTL: carnitine: acylcarnitine translocase
FBS: fetal bovine serum
GO: gene ontology
GSH: glutathione
H_2_O_2_: hydrogen peroxide
HBE: human bronchial epithelial
HE: hematoxylin and eosin
HEPG2: human hepatocarcinoma
KEGG: Kyoto encyclopedia of genes and genomes
MDA: malondialdehyde
MOMP: mitochondrial outer membrane permeabilization
NPs: nanoparticles
O_2_^.-^: superoxide
PFA: paraformaldehyde
PM_2.5_: fine particulate matter
ROS: reactive oxygen species
SE: standard error of the mean
SWCNTs: single-walled carbon nanotubes
TBA: thiobarbituric-acid
