# Supplementary material for: An acetyl-L-carnitine switch on mitochondrial dysfunction and rescue in the metabolomics study on aluminum oxide nanoparticles
Source: Part Fibre Toxicol. 2016 Jan 16;13:4. doi: 10.1186/s12989-016-0115-y (PMC4715336; doi:10.1186/s12989-016-0115-y)
Supplement: Additional file 1: — Supplementary documents Figure S1-3 with figure legend and Tables S1-3. (PDF 793 kb) [file 12989_2016_115_MOESM1_ESM.pdf]

Figure S1 The mitochondrial damages, oxidative stress, lipid peroxidation and apoptosis of HBE cells induced by 12 h $Al_2O_3$ NPs exposure. A) Significant enhancement of apoptosis is only observed in 500 μg/ml $Al_2O_3$ NPs treated HBE cells after 12 h exposure. B) JC-1 staining showed collapse of mitochondrial membrane potential induced by 12 h $Al_2O_3$ NPs treatment. $Al_2O_3$ NPs increase density of green fluorescence and decrease density of red fluorescence in HBE cells. C) After 12h exposure, oxidative stress ROS, $O_2^{\cdot -}$ and MDA , but not $H_2O_2$, are significantly enhanced in HBE cells. Values of apoptosis and oxidative stress assays are presented as mean ± standard error of the mean (SE). Statistically significant differences were determined by one-way ANOVA, followed by Dunnett's multiple comparison tests.

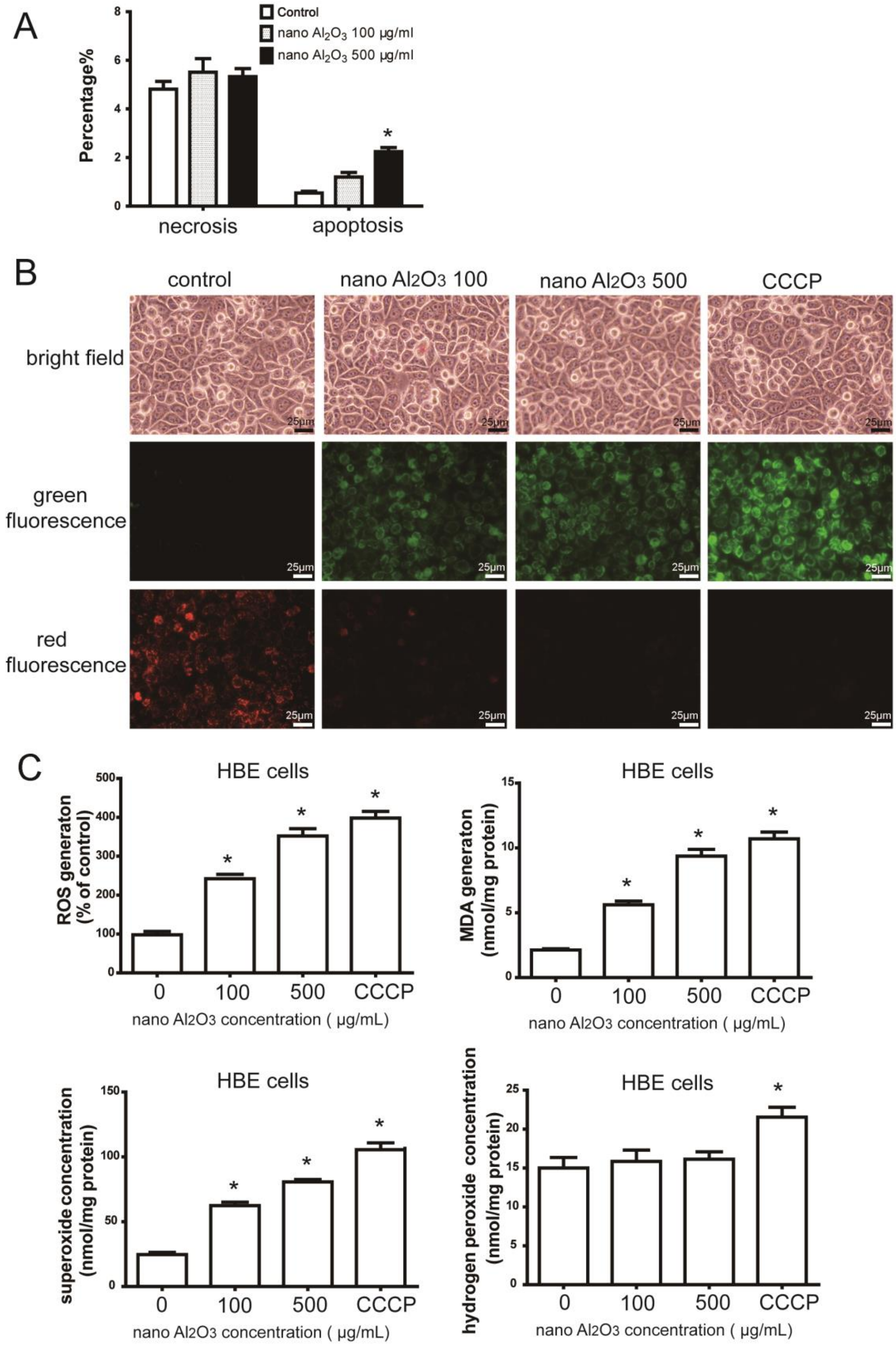
A
Control
nano Al2O3 100 µg/ml
nano Al2O3 500 µg/ml
Percentage%
necrosis
apoptosis
B
control
nano Al2O3 100
nano Al2O3 500
CCCP
bright field
green fluorescence
red fluorescence
25µm
C
HBE cells
ROS generaton (% of control)
MDA generaton (nmol/mg protein)
superoxide concentration (nmol/mg protein)
hydrogen peroxide concentration (nmol/mg protein)
0
100
500
CCCP
nano Al2O3 concentration ( µg/mL)

Figure S2. PCA plot for metabolites of control, 100 and 500 μg/ml $Al_2O_3$ NPs treated HBE cells. The control and two $Al_2O_3$ NPs treated groups distribute separately in two dimension.

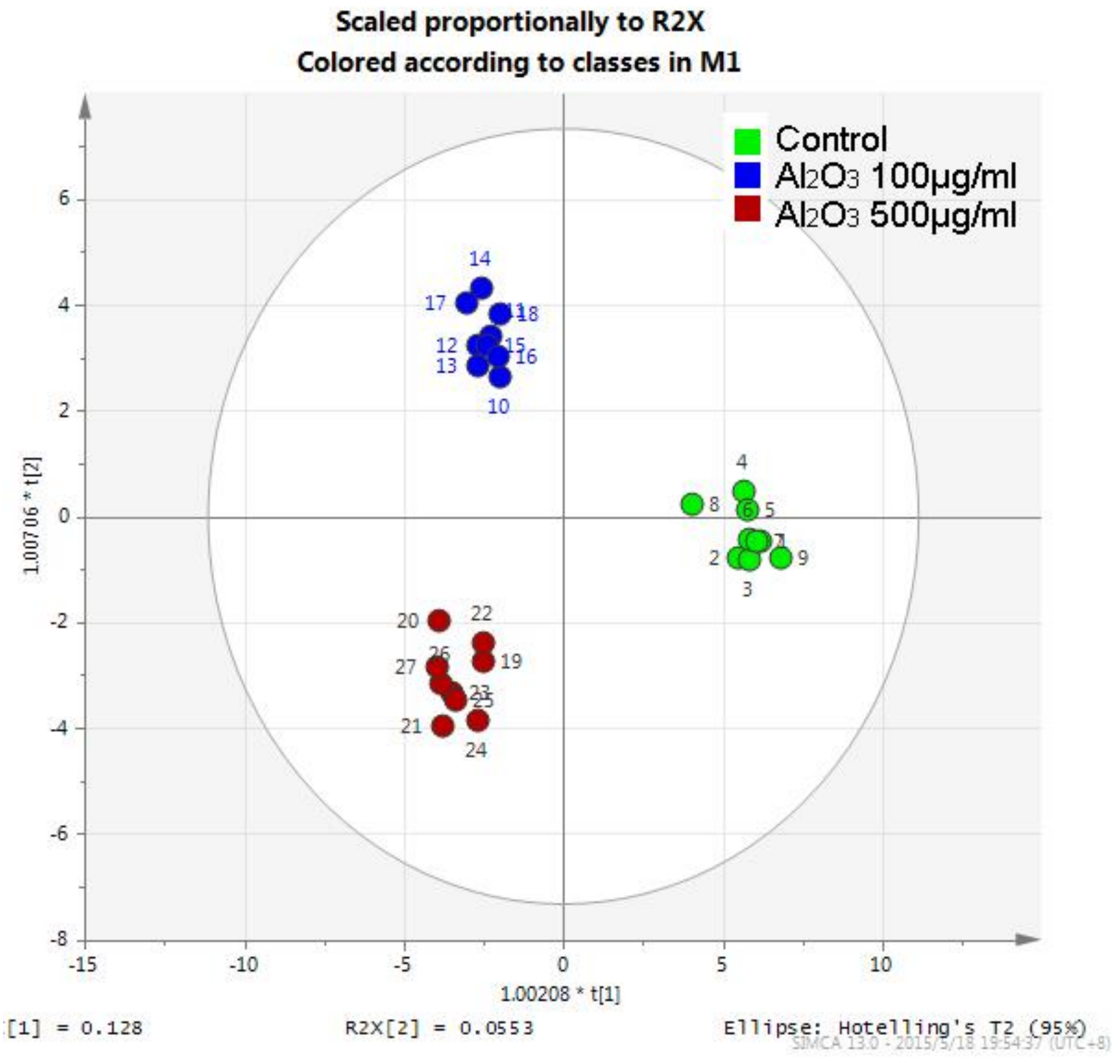

Figure S3. Characterization of $Al_2O_3$ NPs in PBS suspension. A) the average diameter of $Al_2O_3$ NPs suspended in PBS is 64.17 nm, B) the average zeta potential of $Al_2O_3$ NPs suspended in PBS is 37.1 mV.

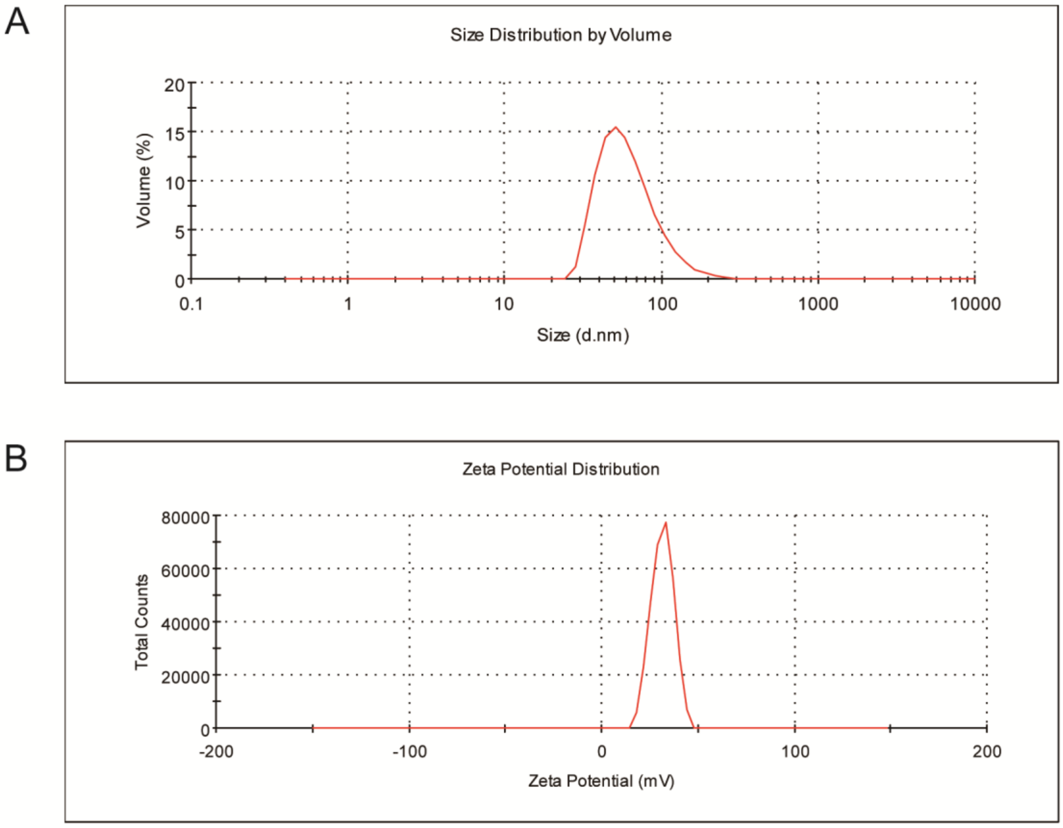

Table S1 Szapiel's scoring of alveolitis [1]

| Score | Grade | Pathological features |
|---|---|---|
| 1 | - | No apparent alveolitis |
| 2 | + | **Mild alveolitis** with pulmonary interstitial edema, inflammatory cell infiltration, and alveolar septum thickening, with lesions only local or limited to the subpleural area, which do not exceed 20% of the lung |
| 3 | ++ | **Moderate alveolitis** with the subpleural area more obvious, with the involved area more than 20% but less than 50% of the lung |
| 4 | +++ | **Severe alveolitis** with lesions more than 50% of the lung, with inflammatory cells inside the alveolar cavity and consolidation changes |

Table S2 The primer sequences of human genes

| | Forward | Reverse |
|---|---|---|
| NDUFA4 | 5’-ATGCTCCGCCAGATCATCG-3’ | 5’-TGCCAGACGCAAGAGATACAG-3’ |
| NDUFA2 | 5’-GCAGCAAGTCGAGGAGTCG-3’ | 5’-CGTTTCTCAATGAAGTCCCTGA-3’ |
| NDUFS4 | 5’-TGCTCGCAATAACATGCAGTC-3’ | 5’-GATCAGCCGTTGATGCCCAA-3’ |
| UQCR11 | 5’-TGCGCAGTGTAGCCGGGTCAGCT-3’ | 5’-GAACTTGACCAGCTCCCGGATGC-3’ |
| COX7B | 5’-CCAGAAATGCACTAAGCAGTCT-3’ | 5’-ACCCATGTAGCAACACAGAAAG-3’ |
| NDUFC2 | 5’-CGGCCTGATTGATAACCTAATCC-3’ | 5’-AAGCTGGCGATGCAAACCA-3’ |
| NDUFA1 | 5’-GCGTACATCCACAGGTTCACT-3’ | 5’-GCGCCTATCTCTTTCCATCAGA-3’ |
| ATP5H | 5’-GCTGGGCGAAAACTTGCTCTA-3’ | 5’-CCAGTCGATAGCTGGTGGATT-3’ |
| COX17 | 5’-TGCGTGTATCATCGAGAAAGGA-3’ | 5’-GCCTCAATTAGATGTCCACAGTG-3’ |
| DLST | 5’-GAACTGCCCTCTAGGGAGAC-3’ | 5’-AACCTTCCTGCTGTTAGGGTA-3’ |
| DS | 5’-TGCTTCCTCCACGAATTTGAAA-3’ | 5’-CCACCATACATCATGTCCACAG-3’ |

Table S3 The primer sequences of mouse genes

| | Forward | Reverse |
|---|---|---|
| NDUFA4 | 5’-TCCCAGCTTGATTCCTCTCTT-3’ | 5’-GGGTTGTTCTTTCTGTCCCAG-3’ |
| NDUFA2 | 5’-TTGCGTGAGATTCGCGTTCA-3’ | 5’-ATTCGCGGATCAGAATGGGC-3’ |
| NDUFS4 | 5’-CTGCCGTTTCCGTCTGTAGAG-3’ | 5’-TGTTATTGCGAGCAGGAACAAA-3’ |
| UQCR11 | 5’-CGTAGTGCTCCAGGGCAGCGGAAC-3’ | 5’-CGGCACCCACAGTGCCCCACATGC-3’ |
| COX7B | 5’-TTGCCCTTAGCCAAAAACGC-3’ | 5’-TCATGGAAACTAGGTGCCCTC-3’ |
| NDUFC2 | 5’-GGCCATGAGCCCTTAAAATTCT-3’ | 5’-CCGTGCAGTAGCCCAACAA-3’ |
| NDUFA1 | 5’-ATGTGGTTCGAGATTCTCCCT-3’ | 5’-TGGTACTGAACACGAGCAACT-3’ |
| ATP5H | 5’-GCTGGGCGTAAACTTGCTCTA-3’ | 5’-CAGACAGACTAGCCAACCTGG-3’ |
| COX17 | 5’-TGCGTGTATCATCGAGAAAGGA-3’ | 5’-GCCTCAATTAGATGTCCACAGTG-3’ |
| DLST | 5’-GGAACTGCCCTCTAGGGAGA-3’ | 5’-GACGCTACCACTGTTAATGACC-3’ |
| DS | 5’-GGACAATTTTCCAACCAATCTGC-3’ | 5’-TCGGTTCATTCCCTCTGCATA-3’ |

## Reference

1. Szapiel SV EN, Fulmer JD,Hunninghake GW,Crystal RG, **Bleomycin-induced interstitial pulmonary diseases in the nude,athymic mouse**. *Am Rev Resp Dis* 1979, **120**:7.
